# *Setaria viridis* as a Model System to Advance Millet Genetics and Genomics

**DOI:** 10.3389/fpls.2016.01781

**Published:** 2016-11-28

**Authors:** Pu Huang, Christine Shyu, Carla P. Coelho, Yingying Cao, Thomas P. Brutnell

**Affiliations:** Donald Danforth Plant Science Center, St LouisMO, USA

**Keywords:** *Setaria viridis*, foxtail millet, bulked segregant analysis, stress tolerance, high-throughput phenotyping, model grass, C4 photosynthesis

## Abstract

Millet is a common name for a group of polyphyletic, small-seeded cereal crops that include pearl, finger and foxtail millet. Millet species are an important source of calories for many societies, often in developing countries. Compared to major cereal crops such as rice and maize, millets are generally better adapted to dry and hot environments. Despite their food security value, the genetic architecture of agronomically important traits in millets, including both morphological traits and climate resilience remains poorly studied. These complex traits have been challenging to dissect in large part because of the lack of sufficient genetic tools and resources. In this article, we review the phylogenetic relationship among various millet species and discuss the value of a genetic model system for millet research. We propose that a broader adoption of green foxtail (*Setaria viridis*) as a model system for millets could greatly accelerate the pace of gene discovery in the millets, and summarize available and emerging resources in *S. viridis* and its domesticated relative *S. italica*. These resources have value in forward genetics, reverse genetics and high throughput phenotyping. We describe methods and strategies to best utilize these resources to facilitate the genetic dissection of complex traits. We envision that coupling cutting-edge technologies and the use of *S. viridis* for gene discovery will accelerate genetic research in millets in general. This will enable strategies and provide opportunities to increase productivity, especially in the semi-arid tropics of Asia and Africa where millets are staple food crops.

## Introduction

Although less prominent than major crops such as rice, maize, and wheat, the polyphyletic millets are important food sources worldwide. Generally, millets are some of the most well-adapted crops to drought, heat, and low nutrient input conditions (Dwivedi et al., 2011; Goron and Raizada, 2015; Saha et al., 2016). Given the increasing global population and decreasing arable lands, the stress tolerant millets are ideal candidates for crop production in climates that are not suitable for major crops. This is especially important for millet-growing developing countries in Asia and Africa. However, common features of millets, including complex polyploid genomes, large plant stature, and long generation times (**Table 1**) hinder both breeding and genetic research (Goron and Raizada, 2015; Saha et al., 2016).

**Table 1:** Comparison of millet species and model grass *Setaria viridis*.

Taxon	Common name	Plant stature^a^	Chromosome no.^b^	Genome size (Mb,1C)	Reference genome	Recent transcriptomic studies	Transformation
*Eleusine coracana*	Finger millet	0.5–1.2 m	4x = 36	1589 (Bennett and Smith, 1976)	In process (ICRISAT)	An et al., 2014; Kumar et al., 2014; Rahman et al., 2014; Singh et al., 2014	Tissue culture (Ceasar and Ignacimuthu, 2011; Ignacimuthu and Ceasar, 2012)
*Panicum miliaceum*	Proso millet	0.2–1.5 m	4x = 36	1017 (Kubešová et al., 2010)		Yue et al., 2016	
*Cenchrus/Pennisetum glaucum*	Pearl millet	up to 3 m	2x = 14	2616 (Bennett and Smith, 1976)	In process (ICRISAT)	Sahu et al., 2012; Choudhary et al., 2015; Kulkarni et al., 2016	Tissue culture (Ramadevi et al., 2014)
*Setaria italica*	Foxtail millet	up to 1.5 m	2x = 18	513 (Bennetzen et al., 2012; Zhang et al., 2012)	Bennetzen et al., 2012; Zhang et al., 2012	Puranik et al., 2013; Yi et al., 2013; Jo et al., 2016	Tissue culture (Wang, 2011)
*Eragrostis tef*	Teff		4x = 40	660 (Bennett and Smith, 1976)	Cannarozzi et al., 2014	Jost et al., 2014	
*Echinochloa esculenta*	Japanese barnyard millet	1–1.5m	6x = 54				
*Echinochloa frumentacea*	Indian barnyard millet	1–1.5m	6x = 54	1296 (Bennett and Smith, 1976)			
*Panicum sumatrense*	Little millet	0.2–1.5 m	4x = 36				
*Setaria viridis*	Green millet	0.1–0.15 m	2x = 18	515 (Li and Brutnell, 2011)	Pre-publication release (phytozome)	Xu et al., 2013; John et al., 2014; Martin et al., 2016	Tissue culture (Brutnell et al., 2010; Van Eck and Swartwood, 2015) and floral-dip (Martins et al., 2015; Saha and Blumwald, 2016)

*^a^Height range from Flora of China (http://www.efloras.org/flora_page.aspx?flora_id=2).*

*^b^Chromosome count show median value from Chromosome count database (http://ccdb.tau.ac.il/) (Rice et al., 2014).*

**FIGURE 1:**
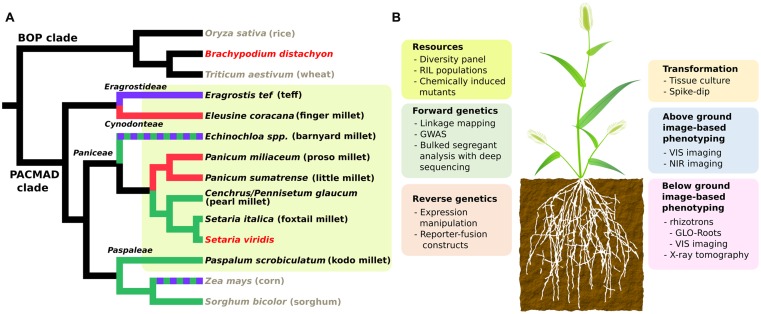
**(A)** Cladogram showing phylogenetic relationships and photosynthetic subtypes of millets and other Poaceae species. Black, gray and red taxa names represent millets, major crops and model grasses, respectively. Millet lineages are also highlighted in yellow. Green, red, purple, and black branch colors represent three subtypes of C_4_ (NADP-malic enzyme, NAD-malic enzyme and phosphoenolpyruvate phosphatase) and C_3_ photosynthesis respectively. Dashed colors represents mixed subtypes of C_4_. Tree topology is extracted from Grass Phylogeny Working Group II (2011). **(B)** Summary chart of available genetic resources and technologies for *Setaria viridis*. RIL, recombinant inbred line; GWAS, genome wide association study; VIS, visual; NIR, near infra-red.

In this review, we discuss the recent development of several genetic and genomic resources in the model grass *Setaria viridis* (green foxtail) and its domesticated relative *S. italica* (foxtail millet). We provide several use cases that demonstrate the value of these resources and their potential to provide new opportunities for breeding and research in millets. *S. viridis* was originally developed as a genetic model for bioenergy feedstocks and panicoid food crops like switchgrass, sorghum, and maize (Doust et al., 2009; Li and Brutnell, 2011; Diao et al., 2014; Brutnell, 2015; Brutnell et al., 2015; Muthamilarasan and Prasad, 2015), and as a model for C_4_ photosynthesis (Brutnell et al., 2010, 2015; Huang and Brutnell, 2016). *S. viridis*, like all millet species, is a member of the PACMAD clade of grasses (**Figure 1**). Previous work in genome organization (Benabdelmouna et al., 2001) and diversity (Huang et al., 2014) shows *S. viridis* is most closely related to and interfertile with foxtail millet. Genetic resources are largely shared between foxtail millet and *S. viridis*, but we emphasize on *S. viridis* in this review because of its nature as an ideal lab organism. Similar to the dicot model *Arabidopsis thaliana, S. viridis* has a short life span (6∼8 weeks under greenhouse conditions), small plant stature (less than 30 cm at maturity) and small diploid genome (∼500 Mb).

## Phylogeny and Photosynthetic Subtypes of Millets

Despite the common small grain nature, millets include grasses from a broad range of phylogenetic clades. We compared the phylogenetic relationship among eight small-seed cereal crops along with other major crops and model species in the Poaceae family based on a previous study (Grass Phylogeny Working Group II, 2011). In this phylogeny, “millet” refers to species from at least four distinct tribes of PACMAD grasses: Paniceae, Paspaleae, Cynodonteae, and Eragrostideae (**Figure 1A**). This polyphyletic nature is also reflected by independent domestications of various millets in different areas of the world (Dwivedi et al., 2011; Goron and Raizada, 2015). Five out of eight species belong to tribe Paniceae, including three major species: pearl millet (*Cenchrus/Pennisetum glaucum*), foxtail millet and proso millet (*Panicum milliacum*), along with the model grass *S. viridis* (**Figure 1A**). Close phylogenetic relatedness generally implies shared genetic mechanism behind complex traits. That is, the more closely related two species are the easier it is to translate genetic discoveries between them. Therefore, compared to other grass models and major crops (**Figure 1A**), *S. viridis* is the most suitable model for most millets from a phylogenetic perspective.

A key feature shared by all millets is C_4_ photosynthesis, regardless of their separate domestication history. Most C_4_ plants, including all the C_4_ grasses utilize specialized bundle sheath and mesophyll cells (Kranz anatomy) to concentrate CO_2_ in the vicinity of ribulose bisphosphate carboxylase/oxygenase. This machinery reduces photorespiration and increases water use efficiency in C_4_ plants (Rawson et al., 1977), especially under drought and heat stress. C_4_ plants also have a better nitrogen use efficiency, namely they require less nitrogen input to achieve similar photosynthetic rates as C_3_ plants (Sage et al., 1987; Sage and Pearcy, 1987a,b). These features of C_4_ correspond nicely with, and likely contribute to the climatic resilience and low soil nutrient demands of millets. Thus, dissecting the genetic basis of C_4_ is an important route to understand the mechanism underlying climatic resilience in millets.

*Setaria viridis* promises to greatly accelerate the pace of discovery in dissecting C_4_ photosynthesis in grasses (Brutnell et al., 2010; Huang and Brutnell, 2016). While genetic screens for C_4_ related mutants in *S. viridis* are currently ongoing, comparative genomics has already provided new insights. For example, Huang et al. (2016) searched for signals of adaptive evolution in two independently evolved C_4_ lineages, *Setaria* and the maize-sorghum clade to identify a candidate gene list for C_4_. The results also indicated a potential for “cross species engineering” of C_4_ transporters. John et al. (2014) showed an 87% correlation between the bundle sheath/mesophyll expression specificity between *S. viridis* and maize, indicating phylogenetically conserved genetic modules controlling C_4_ development. These findings can be generalized to understand C_4_ in other millets. Downstream of candidate gene identification, *S. viridis* as a transformable C_4_ model system also plays a key role in functional characterizations (Martins et al., 2015; Van Eck and Swartwood, 2015; Huang and Brutnell, 2016; Saha and Blumwald, 2016).

## Advances of Forward Genetics in *Setaria* and Other Millets

Classical forward genetic approaches such as linkage and association mapping have been widely applied in most millet species (**Table 1**). However, the lack of high density marker maps is a major limiting factor for the resolution of these applications. Although many quantitative trait loci (QTLs) have been identified for various agronomic traits such as plant height, flowering time, lodging, and drought tolerance (Mauro-Herrera et al., 2013; Parvathaneni et al., 2013; Sato et al., 2013; Babu et al., 2014; Qie et al., 2014; Mauro-Herrera and Doust, 2016; Rajput et al., 2016), the QTL intervals are often large (>1 Mb) and difficult to fine map. A partial solution is to generate high density linkage maps using technologies like genotyping by sequencing (Moumouni et al., 2015; Fang et al., 2016; Rajput et al., 2016), but the ultimate solution is to build high-quality reference genomes. To date, foxtail millet remains the only millet that has a chromosomal scale genome assembly (Bennetzen et al., 2012; Zhang et al., 2012), while *Eragrostis tef* has a draft genome (Cannarozzi et al., 2014), and the genome sequencing of finger millet and pearl millets are still ongoing (**Table 1**). Complete genome sequencing not only enables high density maps (Fang et al., 2016), but also large scale genome wide association studies (GWAS; Jia et al., 2013). Recently, a pre-publication release of an *S. viridis* genome *de novo* assembly became available through phytozome^1^. A panel of accessions in *S. viridis* with a greater genetic diversity than foxtail millet was also assembled for ongoing GWAS (Huang et al., 2014).

Molecular markers are often shared across multiple grass species, further enabling the use of a model species to accelerate gene discovery. For example, Rajput et al. (2014) showed 62% of a total of 339 microsatellite markers are shared between switchgrass and proso millet. One important application of reference genomes is to assist marker development and inform the selection of candidate genes (Parvathaneni et al., 2013). With a closer phylogenetic relationship, more shared synteny and no complicated duplication history, *S. viridis* is generally a better reference than sorghum or maize for both purposes. For example, Hu et al. (2015) examined a diverse panel of pearl millet and showed that shared markers and size of syntenic regions between *Setaria* and pearl millet is more than double of those between sorghum and pearl millet. In addition, *S. viridis* allelic variation can be directly introgressed into foxtail millet through interspecific crosses. Such crosses result in dense molecular markers and additional phenotypic variations, thus greatly facilitating genetic mapping of traits such as flowering time, tillering, and drought tolerance (Mauro-Herrera et al., 2013; Qie et al., 2014; Mauro-Herrera and Doust, 2016).

The short life cycle and small genome of *Setaria* makes it an ideal fit for bulked segregant analysis (BSA). BSA was originally developed for rapid gene mapping in F2 generations (Michelmore et al., 1991). When coupled with deep sequencing technologies, BSA can be conducted faster and without prior knowledge of markers (Takagi et al., 2015). Empirically, the expense of this approach correlates with genome size, and the time to discovery largely depends on the generation time, so this approach is most suitable for model systems. Using this method, Li et al. (2016) mapped a yellow–green leaf mutation in foxtail millet to a chlorophyll biosynthesis related gene *SiYGL1*. Masumoto et al. (2016) mapped a branching panicle mutation, a yield related trait in foxtail millet, to a candidate gene *NEKODE1*. In chemically induced mutants of *S. viridis*, BSA can be expected to define causative mutations to a one to few gene interval within two generations (<7 months). This approach will greatly facilitate genetic dissection of traits such as seed size, inflorescence architecture, flowering time, and climatic resilience (Brutnell, 2015; Brutnell et al., 2015).

## *Setaria viridis* as a Model System to Dissect Gene Function in Millets

Reverse genetics is a powerful tool that enables gene validation and characterization from transcriptomic datasets and/or forward genetics. In light of recent advances in plant biotechnology, reverse genetics is becoming a faster and cheaper routine. There are several important features for a model species to have successful reverse genetic applications: (1) *Plant transformation* is often the most limiting step for most species and therefore it should not be recalcitrant to *Agrobacterium-mediated* transformation (Gelvin, 2003; Ceasar and Ignacimuthu, 2009; Plaza-Wüthrich and Sonia, 2012; Tadele and Plaza-Wüthrich, 2013). (2) *Controlled crosses and prolific seed production* are also essential for rapid genetic analyses (Li and Brutnell, 2011; Brutnell, 2015). (3) *Short life cycle and plant size* is highly advantageous to conduct experiments in controlled environments, and to reduce costs (Brutnell et al., 2010). (4) *Transcriptomic and genomic information* facilitates the selection of candidate genes and inference of potential function based on orthology and/or synteny compared to its relatives (Huang et al., 2016; Huang and Brutnell, 2016). Unfortunately the majority of features are not inherent to most millet species, except in *Setaria*. To date, the techniques and methods of reverse genetics in millets are still very limited, thus a genetic model for millets is greatly needed (Goron and Raizada, 2015).

In recent years, remarkable technical advances were made in the development of resources and techniques for conducting reverse genetics in *S. viridis*. Its inbreeding nature and the ability to perform crosses (Jiang et al., 2013) not only facilitates the generation of homozygous offspring carrying the allele of interest but also enables controlled outcrosses to different populations (i.e., for complementation assays). *Agrobacterium tumefaciens*-mediated gene transfer in *S. viridis* has been successfully developed and first generation events can be produced within 15 weeks (Brutnell et al., 2010; Van Eck and Swartwood, 2015). Alternatively, floral-dip protocols are being developed and would accelerate immensely the pace of gene discovery by reducing the time of callus generation (Martins et al., 2015; Saha and Blumwald, 2016). Together with the rise of genome editing technology using CRISPR/Cas9, model species like *S. viridis* hold the key to accelerate reverse genetic discoveries in C_4_ grasses. It is now possible to generate biallelic mutations and begin downstream gene function characterizations within 1 year, a timeframe which is nearly impossible to match in most crop species. More subtle gene expression manipulations are also possible using modified versions of Cas9 (dCas9) and adding an activator and/or repressor motif to enhance or repress gene expression (Piatek et al., 2015; Zhang et al., 2015). These features and technological advancements in *S. viridis* are especially important for timely characterizations of candidate genes underlying complex traits, including the development of Kranz anatomy and stress tolerance.

Stress tolerance is probably the most explored trait in millets (Charu Lata, 2015; Tadele, 2016). In foxtail millet, several studies have reported on candidate genes regulating drought stress. For example, overexpression of *SiLEA14*, a homolog of the Late embryogenesis abundant (LEA) proteins showed increased salt/drought tolerance and improved growth in foxtail millet (Wang et al., 2014). One important component of abiotic stress responses are Dehydration-Responsive Element Binding (DREB) transcription factors (Li et al., 2014). An abscisic acid (ABA)-responsive DREB-binding protein gene, cloned from foxtail millet (*SiARDP*), was shown to mediate a response that increases tolerance to drought and high salinity stress (Li et al., 2014). Similarly, Lata et al. (2011) identified a *DREB2-like* gene (*SiDREB2*) that is associated with dehydration tolerance and developed an allele-specific marker for tolerant accessions. Technical advances in *Setaria* can also be useful for other millet species for the purposes of functional complementation of orthologous genes. Two recent studies found a NAC and a bZIP transcription factor from finger millet can enhance abiotic tolerance in rice and tobacco, respectively (Babitha et al., 2015; Rahman et al., 2016). As reverse genetic tools advance in *S. viridis*, the pace of gene discovery will also accelerate, enabling the identification of candidate genes that can be introduced into other grasses to confer enhanced abiotic stress tolerance. It will also facilitate the testing of candidate gene function as genes isolated from related millet species can be introduced into *S. viridis* and phenotypes rapidly characterized.

## High-Throughput Phenotyping as a Critical Tool to Advance Millet Research

With the rapid development of genetic tools in *Setaria*, it is critical to have advanced phenotyping techniques to maximize the value of these resources. Automated high-throughput hardware platforms and corresponding software packages are transforming the field of plant-based phenotyping (Yang et al., 2013; Fahlgren et al., 2015b; Rahaman et al., 2015). Here we highlight phenotyping platforms and software packages that have been utilized for *Setaria* and millet research.

Above ground architectural traits such as plant height, biomass and leaf area are important traits for plant breeding (Duvick, 2005). To obtain this information in a high-throughput manner, images are acquired from plants by scanner-based systems or conveyer belt systems under controlled (Fahlgren et al., 2015a; Neilson et al., 2015) or field environments (Vadez et al., 2015). One advantage of these platforms is they allow measurements in a time-dependent manner. For example, Fahlgren et al. (2015a) studied drought responses in *Setaria* using a conveyer belt-based platform. Through image analysis, the authors found that *S. viridis* grows faster and earlier than foxtail millet though they have similar biomass at later time points. *S. viridis* was also found to respond faster to water limitations than foxtail millet. In parallel to 2D images, 3D images can be generated using scanner-based systems. For example, Vadez et al. (2015) used 3D scanning to characterize variations in leaf areas between breeding populations in pearl millet.

Physiological traits can also be measured using specialized imaging systems. For example, using near infra-red (NIR) imaging, Fahlgren et al. (2015a) found strong water content differences between *Setaria* treated with and without water limitation. In addition, fluorescence imaging efficiently measures photosynthesis rate in 2D leaves (Attaran et al., 2014; Cruz et al., 2016), but it is still challenging to measure 3D plants due to confounding height effects (Fahlgren et al., 2015a). Spectroscopy imaging can also be used to examine stress responses (Fahlgren et al., 2015b; Rahaman et al., 2015), but so far this technology has not been utilized in millet research.

Below ground traits contribute greatly to crop performance, but are challenging to image. Therefore, methods for obtaining root images is critical. Rhizotrons are root visualizing systems which hold a thin volume of soil or nutrient substrates between two plastic sheets (Neufeld et al., 1989; Rellán-Álvarez et al., 2015; Passot et al., 2016). This system has been utilized in pearl millets to measure root growth rates (Passot et al., 2016). In *S. viridis*, transgenic lines with a constitutively expressed luciferase reporter provides an imaging system with a cleaner background, known as Growth and Luminescence Observatory for Roots (GLO-Roots; Rellán-Álvarez et al., 2015; Sebastian et al., 2016). Using GLO-Roots, Sebastian et al. (2016) found suppression of crown root growth as a key phenotypic response under water-limiting conditions. To capture 3D structures of root tissues, X-ray tomography has also been utilized in pearl millet, though the system operates at lower throughput (Passot et al., 2016).

As phenotyping systems rapidly develop, it is important to have software packages that can efficiently extract biologically meaningful information from images. Though software such as ImageJ is available (Schneider et al., 2012; Lobet et al., 2013), a new generation of high-throughput, customizable and open-source software is much needed (Fahlgren et al., 2015a; Knecht et al., 2016; Singh et al., 2016). Among them, PlantCV is the first package that has pipelines optimized specifically for *Setaria* (Fahlgren et al., 2015a). Importantly, the small size and rapid growth of *S. viridis* will facilitate its use in both controlled and field-based phenotyping platforms where access to such facilities is often rate limiting.

## Conclusion

Since *Setaria* was initially proposed as a model system for the panicoid grasses (Doust et al., 2009; Brutnell et al., 2010), genetic resources in *Setaria* have been rapidly accumulating. The outstanding model system features of *Setaria* greatly accelerated gene discovery using both classical mapping approaches and new approaches such as BSA coupled with deep sequencing. Availability of transformation techniques along with gene editing technology has also allowed *S. viridis* to be an ideal platform for molecular characterization of gene function. In the meantime, high-throughput phenotyping in *Setaria* has broadened millet research into new dimensions, such as discovery of novel time-dependent traits in plant architecture and physiology.

It is important to note that the use of *S. viridis* is not a substitute for millet research. Rather, *S. viridis* is positioned to become the model for hypothesis testing and genome engineering in order to increase the pace of yield gains and trait enhancements in millets. Usages of this model include but are not limited to, translating mapped genes and QTLs from *Setaria* to other millets, validating candidate genes from other millets in *S. viridis*, and adopting well-established high-throughput phenotyping strategies in *Setaria* to other millets. Finally, fundamental understandings of important complex traits such as C_4_ photosynthesis and stress tolerance in *Setaria* will greatly benefit studies of these commonly shared features in all millets, and create new opportunities to accelerate millet breeding and genetic engineering.

## Author Contributions

PH and TB conceived of the manuscript. PH, CS, CC, YC, and TB wrote the manuscript. All authors read and approved the final manuscript.

## Conflict of Interest Statement

The authors declare that the research was conducted in the absence of any commercial or financial relationships that could be construed as a potential conflict of interest.

## Footnotes

1 http://phytozome.jgi.doe.gov/
